# Metabolic and Transcriptomic Profiling Reveals Etiolated Mechanism in Huangyu Tea (*Camellia sinensis*) Leaves

**DOI:** 10.3390/ijms232315044

**Published:** 2022-11-30

**Authors:** Xin Mei, Kaikai Zhang, Yongen Lin, Hongfeng Su, Chuyuan Lin, Baoyi Chen, Haijun Yang, Lingyun Zhang

**Affiliations:** 1 College of Horticulture, South China Agricultural University, Guangzhou 510642, China; 2 Center for Basic Experiments and Practical Training, South China Agricultural University, Guangzhou 510642, China

**Keywords:** *Camellia sinensis* cv. Huangyu, yellowing, leaf color, chlorophyll, biological mechanism

## Abstract

Leaf color is one of the key factors involved in determining the processing suitability of tea. It relates to differential accumulation of flavor compounds due to the different metabolic mechanisms. In recent years, photosensitive etiolation or albefaction is an interesting direction in tea research field. However, the molecular mechanism of color formation remains unclear since albino or etiolated mutants have different genetic backgrounds. In this study, wide-target metabolomic and transcriptomic analyses were used to reveal the biological mechanism of leaf etiolation for ‘Huangyu’, a bud mutant of ‘Yinghong 9’. The results indicated that the reduction in the content of chlorophyll and the ratio of chlorophyll to carotenoids might be the biochemical reasons for the etiolation of ‘Huangyu’ tea leaves, while the content of zeaxanthin was significantly higher. The differentially expressed genes (DEGs) involved in chlorophyll and chloroplast biogenesis were the biomolecular reasons for the formation of green or yellow color in tea leaves. In addition, our results also revealed that the changes of DEGs involved in light-induced proteins and circadian rhythm promoted the adaptation of etiolated tea leaves to light stress. Variant colors of tea leaves indicated different directions in metabolic flux and accumulation of flavor compounds.

## 1. Introduction

Tea (*Camellia sinensis*) is an important beverage plant. The color of tea leaves not only decides the appearance of the made tea, but also relates to the physical and chemical quality, which further affects the economic benefits. In terms of production, the tea tree resources with leaf-color variation are not only unique in appearance but will also offer a unique sensory quality. According to the colors of buds and leaves, leaf-color mutants of tea trees can be divided into three categories: albino, etiolation (yellowing), and variegation. Furthermore, according to their regreening mechanisms, these mutants can also be grouped as light- and temperature-sensitive types. Although they are different in appearance, the change in leaf color is mainly due to abnormal chloroplast development and blocked chlorophyll synthesis, which will then directly affect photosynthesis and the accumulation of metabolites in tea leaves, resulting in different tea qualities [1]. Interestingly, comparing these tea leaves with those of normal color, most of the teas made from albino or etiolated fresh tea leaves were lighter in color and fresher in taste, which is closely related to the change in the chemical components of fresh leaves. For example, the light-sensitive new shoots of albino tea trees grew yellow and white in strong light, which led to lower tea polyphenols and higher amino acids, and the finished tea tasted fresher [2,3]. However, light-sensitive new shoots of albino tea cultivars have weak resistance and are easily damaged by high temperatures and strong light at the seedling stage [4]. Thus, revealing the molecular biological mechanism of etiolated mutants is meaningful for light-sensitive tea cultivars to promote their development and enhance their resistance to environmental stress.

Studies have shown that the albino or etiolated mutants may be caused by a variety of factors. One of the most critical reasons is the decrease of chlorophyll anabolism in the etiolated mutant. The biochemical reactions of chlorophyll synthesis are catalyzed by a series of enzymes. Mutations in any gene involved in chlorophyll synthesis would lead to the reduction or loss of enzyme activities, which in turn affects the synthesis of related proteins, thus repressing the synthesis of chlorophyll [5]. As a result, the content of chlorophyll was reduced, the development of chloroplasts was repressed, and the phenotype of the leaf color presented as yellow or albino [6]. For example, the content of chlorophyll in the leaf-etiolated mutant 812HS of rice is significantly reduced. On the other hand, the reduction of thylakoid membrane protein complexes affects the harvest and transmission of light [7]. Some studies have found that the expression of light-harvesting complex (LHC-II) in the etiolated mutant decreased moderately, the light-harvesting antenna of PS I was severely damaged, and the expression of PS II core protein and ATP synthase increased [8]. In an etiolated tea plant (‘Huangjinya’), chlorophyll synthesis in etiolated leaves was blocked at the coprogen III→proto IX stage [9]. The formation of thylakoid and grana in the chloroplast of yellow leaves was repressed, and the content of chlorophyll decreased significantly. It was speculated that the content of chlorophyll in etiolated leaves was deficient, and total carotenoids, violaxanthin, and lutein were maintained at high levels, which was a critical reason for ‘Huangjinya’ forming its yellow leaf color [10]. The down-regulated expression of protoporphyrinogen oxidase and protox/PPOX (CsPPOX1) regulated by strong light was the main reason for the blocked chlorophyll synthesis in ‘Huangjinya’ [11]. In variegated leaves, the high expression of the chlorophyllase (CLH) gene and higher enzyme activity promoted chlorophyll degradation, while the low expression of light-harvesting chlorophyll a/b protein (Lhcb1, Lhcb3) and protochlorophyllide oxidoreductase (POR) in chlorophyll synthesis might lead to the lower chlorophyll content in variegated leaves [12].

Since the formation of different leaf colors is mostly caused by natural mutation, previous research on leaf-color mutants and tea quality components mainly focused on tea plants with different genetic backgrounds [1,2,13,14]. Few literatures have compared characteristics of tea with the same genetic background. ‘Yinghong 9’ (*Camellia sinensis* var. assamica cv. Yinghong No. 9) is a clonal cultivar selected from the large-leaf species in the Yunnan province of China. It has been widely planted in southern China in recent years. Researchers accidentally discovered its etiolated bud-mutant, namely ‘Huangyu’. Previous studies have analyzed the differences between the etiolated mutant and ‘Yinghong 9’ in leaf colors, theanine formation, proteomics, and so on, and explained the possible causes of leaf-color variation and the accumulating mechanism of amino acids [1,15]. However, the molecular mechanism of the leaf-color mutation remains unclear.

In this study, biochemical, metabolome, and transcriptome analysis were used to study the etiolated mechanism of ‘Huangyu’. The mechanism of leaf-color formation was discussed between ‘Huangyu’ and ‘Yinghong 9’ according to metabolic profiling. The differentially expressed genes (DEGs) and main regulatory transcription factors involved in leaf-color formation were identified by transcriptome analysis. The current study provides a theoretical basis for the application of etiolated tea cultivars.

## 2. Results

### 2.1. Phenotype and Pigment Content

**Figure 1:**
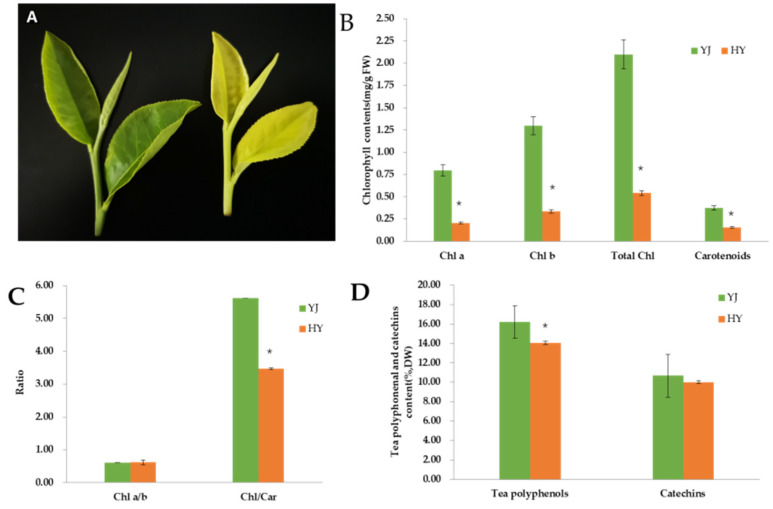
Phenotypic characteristics of Yinghong 9 (**left**) and Huangyu (**right**) (**A**) and pigment contents (**B**–**D**) of ‘Yinghong 9’ and ‘Huangyu’. The data are presented as the mean ± standard deviation (*n =* 3). * indicates significant difference (*p* < 0.05) between ‘Yinghong 9’ and ‘Huangyu’. YJ is ‘Yinghong 9’, HY is ‘Huangyu’.

As shown in Figure 1A, the leaf color of ‘Yinghong 9’ is normal green, while the leaves of its mutant ‘Huangyu’ are golden yellow. However, from the phenotypic characteristics, there was no significant difference in bud type, leaf length, or leaf thickness between the two groups. The result suggests that changes in leaf color have no effects on leaf morphology. Consistent with the leaf phenotype, the contents of chlorophyll a, chlorophyll b, and carotenoids in ‘Yinghong 9’ were higher than those in ‘Huangyu’. Moreover, the total amount of tea polyphenols and catechins in the leaves of ‘Huangyu’ was also lower than that in ‘Yinghong 9’, but the difference in the total content of catechins did not have a significant difference. These results are consistent with the previous studies, that is, the total amount of chlorophyll in leaves of etiolated tea cultivars is always lower than that in normal leaves, and the total amount of tea polyphenols and catechins were also slightly lower than that in green leaves. The above results also indicated that the reduction of pigment compounds in the leaves of mutants, especially the Chl/Car ratio, might be responsible for leaf yellowing.

### 2.2. Differential Metabolites in ’Yinghong 9’ and ’Huangyu’

**Figure 2:**
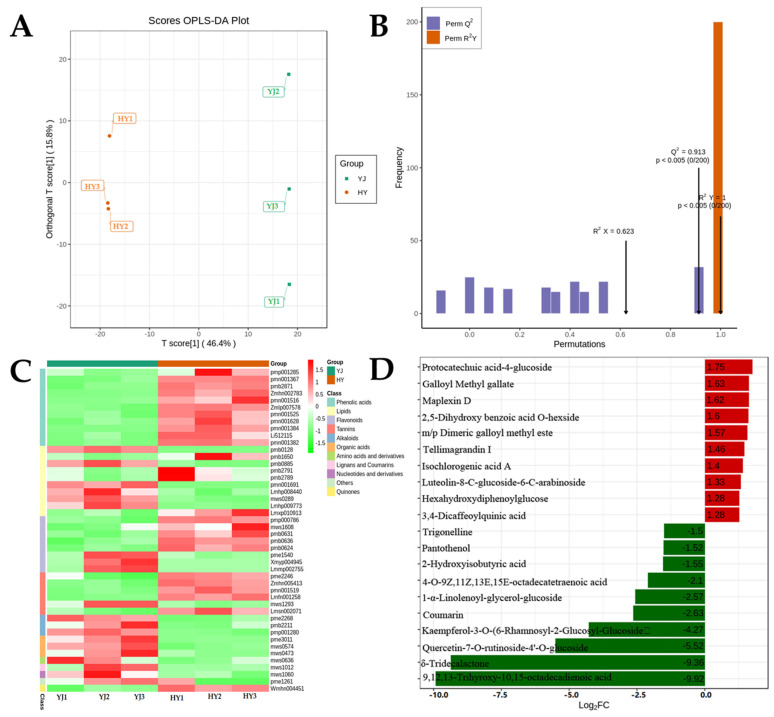
Metabolome quality and differential metabolites analysis: (**A**) OPLS-DA, (**B**) Permutation test for OPLS-DA model, *Q*^2^ > 0.5, R^2^ > 0.5, *p* < 0.005; (**C**) Type of differential metabolites and expression of up-/down-regulation; and (**D**) Differential metabolites of Top 20 FC (fold change of YJ/HY) between ‘Yinghong 9’ (YJ) and ‘Huangyu’ (HY)). YJ was ‘Yinghong 9’ leaves, HY was ‘Huangyu’ leaves. pmn001519, Galloyl Methyl gallate; Lmfn001258, Maplexin D; Zmhn005413, m/p Dimeric galloyl methyl ester; Lmsn002071, Tellimagrandin I; pme2246, Ellagic acid; mws1293, Theaflavin; Wmhn004451, 1,4,8-Trihydroxynaphthalene-1-O-[6′-O-(3′,4′,5′- trimethylbenzoyl)] glucoside; pmn001367, Protocatechuic acid-4-glucoside; pmb2871, 2,5-Dihydroxy benzoic acid O-hexside; pmn001382, Isochlorogenic acid A; pmn001628, Hexahydroxydiphenoylglucose; Li512115, 3,4-Dicaffeoylquinic acid; pmn001525, 3,5-Di-O-galloylshikimic acid; Zmlp007578, 3,3′,4-Trimethoxy ellagic acid; pmp001285, Phthalic anhydride; Zmhn002783, 5-O-Galloylshikimic acid; pmn001516, 5-Galloylshikimic acid; pmn001384, Isochlorogenic acid C; pme1261, Pantothenol; mws0473, 2-Methylsuccinic acid; pme3011, γ-Aminobutyric acid; mws0574, 2-Hydroxyisobutyric acid; mws1060, 9-(Arabinofuranosyl)hypoxanthine; pmb2789, 13S-Hydroperoxy-6Z,9Z,11E-octadecatrienoic acid; Lmxp010913, 1-(9Z-Octadecenoyl)-2- (9-oxo-nonanoyl)-sn-glycero-3-phosphocholine; pmb2791, 9-HpOTrE; pmb1650; Octadeca-11E,13E,15Z-trienoic acid; Lmhp008440, LysoPE 15:1; mws0289, LysoPE 18:1; pmb0885, 4-O-9Z,11Z,13E,15E-octadecatetraenoic acid; Lmhp009773, 1-α-Linolenoyl- glycerol-glucoside; pmb0128, δ-Tridecalactone; pmn001691, 9,12,13-Trihyroxy-10,15-octadecadienoic acid; mws1012, Coumarin; pmb0631, Luteolin-8-C-glucoside-6-C-arabinoside; pmb0624,Luteolin-6-C-glucoside-7-O-glucoside; pmp000786, Eupatorin; pmb0636, Luteolin-8-C-glucoside-7-O-arabinoside; mws1608, Luteolin-6-C-glucoside (Isoorientin); pme1540, Isorhamnetin-3-O-neohesperidoside; Xmyp004945, Camelliaside A; Lmmp002755, Quercetin-7-O-rutinoside-4′-O-glucoside; mws0636, Phe-Phe; pmb2211, Cocamidopropyl betaine; pmp001280, 3-{[(2-Aminoethoxy)(hydroxy)phosphoryl] oxy}-2-hydroxypropyl-9,12-octadecenoate; pme2268, Trigonelline.

In this study, a total of 609 metabolites were identified in ‘Yinghong 9’ and ‘Huangyu’ by wide-targeted metabolomics. The results of OPLS-DA analysis showed that the metabolite distributions of ‘Yinghong 9’ and ‘Huangyu’ were clearly separated (Figure 2A,B). A total of 46 differential metabolites were screened (FC ≥ 2 or ≤ 0.5, *p*-value < 0.05, VIP > 1.0), of which 26 were up-regulated and 20 were significantly down-regulated (Supplementary Table S3A). Among the differential metabolites, alkaloids and organic acids were all down-regulated, while almost all phenolic acids were up-regulated. However, four glycosides including luteolin-6-c-glucoside (isoorientin) were up-regulated in flavonoids, and three important compounds of flavonols were down-regulated, especially quercetin-7-O-rutinoside-4′-O-glucoside which showed the highest down-regulation level in ‘Huangyu’ (Log_2_FC = −5.516) (Supplementary Table S3B). The major differences between ‘Huangyu’ and ‘Yinghong 9’ were phenolic acids. The content of phenolic acids in ‘Huangyu’ was higher than that in ‘Yinghong 9’, and the changes in flavonoids content coincided well with that of phenolic acids content in ‘Yinghong 9’ and ’Huangyu’ (Figure 2C). Among the 26 up-regulated metabolites, protocatechuic acid-4-glucoside showed the highest increased level in ‘Huangyu’, increasing about 1.75-fold (Figure 2D). Since flavonoids and phenolic acids are the critical flavor compounds in finished tea, our results revealed that most of the top 10 up-regulated metabolites were phenolic compounds (phenolic acids or tannins). By comparison, most of the top 10 down-regulated metabolites were lipids (high content in ‘Yinghong 9’). The difference was possibly due to metabolic flux in the two cultivars because of their different leaf colors.

### 2.3. Carotenoid Identification and Quantification

**Figure 3:**
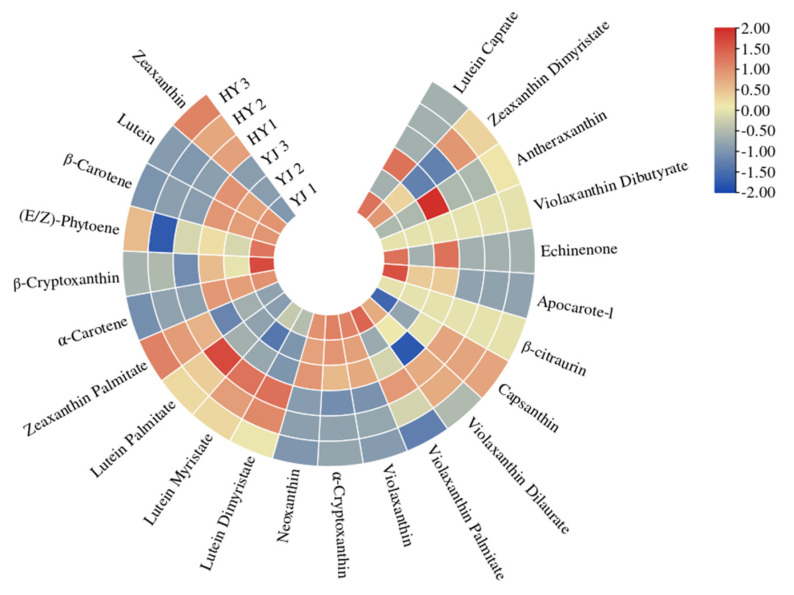
Heat map of carotenoids in ‘Yinghong 9’ and ‘Huangyu’. Red and blue indicate higher and lower abundances, respectively. YJ was ‘Yinghong 9’; HY was ‘Huangyu’.

In the previous study, the content of total carotenoids was higher in normal green leaves of the cultivar ‘Yinghong 9’ than that in ‘Huangyu’. In this study, qualitative and quantitative analyses of carotenoids in ‘Yinghong 9’ and its etiolated mutant were conducted by using UHPLC-APCI-MS/MS. A total of 23 carotenoids were identified in ‘Yinghong 9’ and ‘Huangyu’, including *α*-carotene, *β*-carotene, lutein, zeaxanthin, antheraxanthin, violaxanthin, neoxanthin, apocarote, *α*-cryptoxanthin, *β*-cryptoxanthin, capsanthin, (E/Z)-phytoene, apocarotenal, zeaxanthin, lycopene, and so on. Among them, *α*-carotene, *β*-carotene, lutein, and zeaxanthin were the main components in ‘Yinghong 9’ and ‘Huangyu’. While antheraxanthin, violaxanthin, neoxanthin, apocarote, *α*-cryptoxanthin, *β*-cryptoxanthin, capsanthin, (E/Z)-phytoene, apocarotenal, zeaxanthin, lycopene, violaxanthin, and neoxanthin were present at low levels in ‘Yinghong 9’, and were even not detected in ‘Huangyu’ (Supplementary Table S4). Compared with ‘Huangyu’, the contents of lutein, (E/Z)-phytoene, *α*-carotene, *β*-carotene, *β*-cryptoxanthin, and neoxanthin were significantly higher in ‘Yinghong 9’. However, the content of zeaxanthin was significantly higher in ‘Huangyu’ (Figure 3 and Supplementary Table S4).

### 2.4. Transcriptome Sequencing and Annotation

Altogether there were six samples subjected to RNA-seq analysis, and a total of 41.66 Gb clean data was obtained. The clean data of each sample reached 5.88 Gb, and Q30 percentage was over 93.99%. The clean reads of each sample were mapped to the tea tree genome (http://tpdb.shengxin.ren/, accessed on 29 March 2022), and the alignment efficiency ranged from 81.50% to 82.42%. Based on the alignment results, a total of 6696 were functionally annotated (Supplementary Table S5). Through the differential expression analysis between two cultivars, a total of 1009 DEGs were identified (FDR < 0.01, FC ≥ 1) (Supplementary Table S6A).

### 2.5. Screening of DEGs in ’Yinghong 9’ and ‘Huangyu’

**Figure 4:**
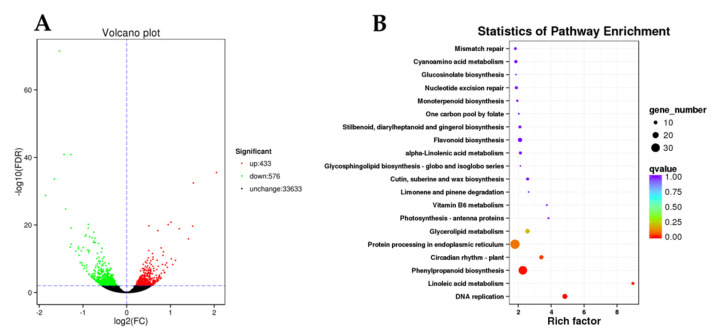
Volcano plot of differentially expressed transcripts (**A**) and KEGG enrichment analysis of DEGs (**B**) in ‘Yinghong 9’ and ‘Huangyu’.

The DEGs were screened by KOG and KEGG enrichment analyses, and results showed that 1009 candidate DEGs were enriched in 107 metabolic pathways, of which four pathways were significantly enriched (corrected *p*-value < 0.05) (Supplementary Table S7). Among which the primary enriched pathways were DNA replication, linoleic acid metabolism, phenylpropanoid biosynthesis, circadian rhythm-plant, protein processing in endoplasmic reticulum, glycerolipid metabolism, flavonoid biosynthesis, cutin, suberine and wax biosynthesis, photosynthesis-antenna proteins, alpha-linolenic acid metabolism, stilbenoid, diarylheptanoid, and gingerol biosynthesis (Figure 4 and Supplementary Table S7).

The results of KEGG analysis showed that a total of seven genes in the linoleic acid metabolism pathway had been significantly up-regulated, among which were lipoxygenase or its family genes. Since lipoxygenase can catalyze the oxidation of corresponding substrates, the substances might lead to the oxidative decomposition of chlorophyll; therefore, it might be directly related to the chlorophyll metabolism in the two cultivars. A total of fifteen genes in the DNA replication metabolic pathway shown were significantly up-regulated (Figure 4), including multiple family genes involved in the light response. In the photosynthesis-antenna proteins metabolic pathway, three chlorophyll a-b binding protein (LHCII type 1) genes (CSS0013089, CSS0017825, CSS0039893) with higher expression levels were screened, and the expression levels of these genes in ‘Huangyu’ were lower than those in ‘Yinghong 9’ (Supplementary Table S8). Thirty-six significantly enriched DEGs were screened involved in the phenylalanine metabolism and flavonoid metabolism pathways, including two FLS, one DFR, and two LAR. Interestingly, all flavonoid pathway genes were up-regulated in the etiolated mutant (Huangyu); while, in the phenylalanine metabolism pathway, six genes were down-regulated, whereas all the remaining thirty genes were up-regulated (Supplementary Table S6B). In the circadian rhythm-plant metabolic pathway, twelve DEGs were screened, among which ten genes were down-regulated in the etiolated mutant (Huangyu). In addition, three DEGs involved in the chlorophyll synthesis pathway were screened, namely HEME (heme A synthase, CSS0016239), GUN5 gene (magnesium-chelatase subunit ChlH, CSS0016317), and CAO gene (chlorophyllide a oxygenase, CSS0032816) (Supplementary Table S8); the expression levels of these genes in the etiolated mutant were significantly lower than those of normal green leaves (Yinghong 9). In the chlorophyll degradation pathway, chlorophyllase (CLH, CSS0004684) was also the main differentially expressed gene, and the expression level of CLH in the etiolated mutant (Huangyu) was significantly higher than that of ‘Yinghong 9’. Due to CLH being involved in chlorophyll degradation, the expression level of the CLH in albino tea is usually higher than in normal green leaf tea [12].

### 2.6. Identification of Differentially Expressed Transcription Factors

A total of 100 transcription factors were screened from DEGs, among which the top 15 highly expressed transcription factors (FPKM > 80) were involved in light-responsive and photomorphogenesis functions (Supplementary Table S9), such as HY5 (ELONGATED HYPOCOTYL5), RADIALIS-like, MYB75, MYB12, MYB123, and so on. Some transcription factors were involved in plant hormones, such as ethylene-responsive transcription factor RAP2-2, auxin-responsive protein IAA9, ETHYLENE INSENSITIVE 3-like 1 protein, and so on. HY5 is a transcription factor that plays a positive regulatory role in the light signaling pathway, and is involved in the regulation of biological processes such as photomorphogenesis, chloroplast development, as well as pigment accumulation. HY5 can also promote the transport of photosynthetic products in the shoots of plants. Since the expression of HY5 was down-regulated in ‘Huangyu’, it might be related to its lower chlorophyll content and lower photosynthetic capacity. In addition, among the highly expressed transcription factors, there is ethylene-insensitive 3 (EIN3), which can participate in the regulation of anthocyanin synthesis in *Arabidopsis*. The MYB14 transcription factor is involved in the regulation of procyanidin accumulation [16]. MYB75-like or MYB75/PAP1 is a class of R2R3-MYB transcription factors involved in the regulation anthocyanin biosynthesis in *Arabidopsis*. Overexpression of the MYB75 gene (*Ox*MYB75) can lead to the excessive accumulation of anthocyanins in *Arabidopsis* leaves, roots, stems, and flowers. The MYB75 gene not only is involved in regulation of the anthocyanin synthesis pathway but also participates in the regulation of the flavonol synthesis pathway. Overexpression of MYB75 can increase anthocyanin and flavonol levels in *Arabidopsis thaliana* and this increases resistance to omnivorous insects [17]. Since the expression levels of the above-mentioned R2R3-MYB transcription factors were down-regulated in ‘Huangyu’, it implied that the photosynthetic capacity was lower than that of ‘Yinghong 9’, which led to less accumulation of anthocyanins and tea polyphenols in ‘Huangyu’.

### 2.7. Validation of Gene Expression Levels by qRT-PCR

**Figure 5:**
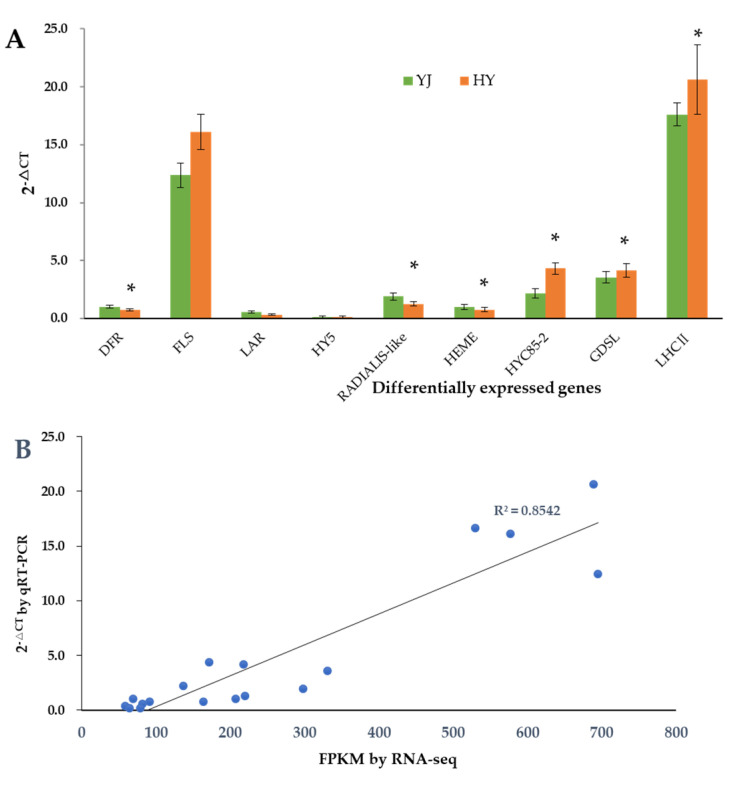
Quantitative real-time polymerase chain reaction (qRT-PCR) validation of the DEGs. (**A**) Relative expression levels of genes. * indicates significant difference (*p* < 0.05) between ‘Yinghong 9’ and ‘Huangyu’. (**B**) Correlation analysis between the FPKM value and qRT-PCR results. All data are shown as mean ± SE (*n =* 3). YJ was ‘Yinghong 9’ fresh leaves, HY was ‘Huangyu’ fresh leaves.

To confirm the accuracy of the RNA-seq results, nine key DEGs were selected to be further validated by quantitative real-time polymerase chain reaction (qRT-PCR). The expression profiles of most of the selected genes were similar in RNA-seq and qRT-PCR analyses, and the correlation coefficient between the qRT-PCR and FPKM value by RNA seq was 0.8542 (Figure 5). These results demonstrated that the transcriptome data were reliable.

## 3. Discussion

The color of tea leaves plays an important role in sensory quality and processing suitability in tea cultivars. The mechanisms of leaf color formation have been studied on some albino tea cultivars. Unfortunately, the yellowing mechanisms of leaves have been studied on etiolated tea always with different genetic backgrounds. While ‘Huangyu’, the bud mutation of ‘Yinghong 9’, can be used to study the molecular mechanism of leaf-color formation because of their same genetic background. Previous studies revealed that ‘Huangyu’ was also the light-sensitive etiolated cultivars. However, the molecular mechanism of its etiolated leaf color is still unclear. In the current study, our primary goal was to elucidate the metabolic characteristics of the ‘Huangyu’ etiolated leaf and the molecular mechanisms responsible for the leaf color changes using metabolome and RNA-seq analysis.

### 3.1. The Leaf Phenotype Is Associated with Content of Chlorophyll and Carotenoid in ‘Yinghong’ 9 and ‘Huangyu’

Chlorophyll is a crucial pigment for plant leaf-color formation and photosynthesis [12]. Previous studies have shown that the formation of tea leaves’ color from white to purple may be due to changes in different pigments and their ratios. In general, the greening of leaves is mainly due to higher chlorophyll contents, whereas the albino-induced white and yellow leaves are mainly due to lower chlorophyll contents as well as certain concentrations of carotenoids which are dominant in leaf-color formation [18,19]. As mentioned above, carotenoids, as important pigments in green leaves, can protect chlorophyll from photo-oxidation by scavenging various reactive oxygen radicals. The lower chlorophyll content in yellow leaves is due to the blockage of chlorophyll synthesis or by an increase in the chlorophyll degradation rate. Some studies speculated that the leaf color changes in ‘Huangjinya’ are largely determined by the combined effects of flavonoid and carotenoid biosynthesis [20].

One of the reasons that plants produce yellow leaves is that carotenoid biosynthesis is repressed, and the reduction of carotenoids contents leads to photooxidative damage which occurs in the chloroplasts under strong light or normal light, which results in defective chloroplast development [21]. Some studies believed that carotenoids could harvest and transmit light energy in photosynthesis and had certain reducibility to protect chlorophyll from oxidative damage by light [22]. When carotenoid synthesis was blocked, it would also lead to a loss of protection of chlorophyll, eventually resulting in abnormal chloroplast development [23]. Some studies also believed that the synthesis of chlorophyll in yellow leaves was repressed, and the synthesized chlorophyll would also be degraded [24]. For temperature-sensitive tea cultivars, the biosynthesis of chlorophyll and carotenoids is inhibited under low-temperature conditions, which decreases in the levels of chlorophyll and carotenoids, and eventually leads to the production of etiolated mutants [25]. The reason may be that the biodegradation rate of chlorophyll and other pigments is much faster than their biosynthesis rate. While, for light-sensitive tea mutants, strong sunlight up-regulated the expression of genes involved in the carotenoid pathway, resulting in the accumulation of carotenoids, and strong sunlight induced hypoplasia of chloroplasts by suppressing the development of grana stacking and thylakoids, so the lack of chlorophylls and the accumulation of carotenoids were considered to cause the yellow color formation in etiolated shoots [10]. Similar research results have been reported in some etiolated mutants, for example, ‘Anji baicha’ [26], ‘Huabai 1’ [13], ‘Xiaoxueya’ [27], ’Yujinxiang’ [28], ‘Huangjinya’ [20], and ‘Menghaihuangya’ [29]. The current study results showed that the total chlorophyll content of normal green leaves of ‘Yinghong 9’ was significantly higher than that of yellow leaves of ‘Huangyu’ (Figure 1B).

In this study, the changes of the content of tea polyphenols, chlorophyll, and carotenoids were similar in the two cultivars. Compared with ‘Yinghong 9’, the contents of zeaxanthin and zeaxanthin palmitate in ’Huangyu’ leaves were significantly higher. The contents of *α*-carotene, (E/Z)-phytene, *β*-cryptoxanthin, *β*-carotene, and neoxanthin were higher in ‘Yinghong 9’ leaves (Figure 3). Since zeaxanthin is an important metabolite of *β*-carotene in plants, and *β*-carotene is catalyzed by *β*-carotene hydroxylase (BCH) to generate zeaxanthin [30]. Silencing the BCH gene led to an increase in *β*-carotene and carotenoids levels in potato tubers but decreased zeaxanthin levels [31]. The results are consistent with findings of previous research in ‘Huangjinya’ and ‘Yujinxiang’ yellow leaves, which are light-sensitive tea cultivars, but with slight differences in carotenoid levels, such as the contents of zeaxanthin, *β*-carotene, and xanthophyll are higher in ‘Huangjinya’, while the contents of lutein, carotene, cryptoxanthin, and violaxanthin are higher in ‘Yujinxiang’ [32]. Zeaxanthin, which is produced from violaxanthin, has an antioxidant effect to protect thylakoid membranes from photooxidative stress [33], and zeaxanthin can be induced and accumulated in plants under different abiotic stresses such as excessive light, drought, and low humidity [34]. It has been observed in other etiolated mutants that the content of zeaxanthin is 2–4-fold higher than that of normal green leaf tea trees (Fuding Dabaicha) [24]. The mechanism might be that the higher zeaxanthin level in etiolated tea might be very helpful to maintain normal photosynthetic capacity and efficiency under excess light and low temperature stress [35]. The similar results have been demonstrated in several etiolated tea plant cultivars [36]. All in all, despite the higher total carotenoid levels in ‘Yinghong 9’, chlorophyll may conceal the coloration of carotenoids, and result in the formation of green in the normal ‘Yinghong 9’ leaves. As for ‘Huangyu’, having a lack of chlorophyll in the leaves, and the coloring of the leaves was mainly attributed to the content of carotenoids.

### 3.2. Changes in the Expression of Genes Involved in Chlorophyll Synthesis and Chloroplast Biogenesis Led to Leaf Color Mutation

#### 3.2.1. Key Genes Involved in Chlorophyll Synthesis Pathway Were Down-Regulated in Huangyu

The chlorophyll biosynthesis pathway includes the following three stages, namely the synthesis of chlorophyll a (Chl a), the transformation of chlorophyll a/chlorophyll b (Chl b), and the degradation of chlorophyll a [37,38]. This pathway requires 15 enzymes encoded by 27 genes to participate in the transformation from the formation of L-glutamyl-tRNA (Glu-tRNA) to Chl a and Chl b [39,40]. The biosynthesis of chlorophyll is affected by environmental factors such as light intensity, photoperiod, and light quality. CAO, encoding chlorophyllide a oxygenase, is responsible for Chl b biosynthesis. Abnormal function of this enzyme can lead to a rapid decrease in Chl b content, or even failure to synthesize Chl b, resulting in leaf yellowing; the expression of CAO is regulated by light conditions [41,42]. Studies have found that the expression levels of CAO in *Arabidopsis* was very low under lower light conditions, but significantly increased under higher light conditions [43]; while under higher light conditions, early light-induced proteins (ELIPs) increase rapidly, thereby inhibiting glutamyl–tRNA reductase (GluTR) and the Mg-chelatase subunits CHLH and CHLI to prevent the accumulation of free chlorophyll, and hence prevent photooxidative stress [44]. In addition, GUN4-porphyrin complexes bind the ChlH/GUN5 to promote chlorophyll biosynthesis by activating Mg-chelatase, and the role of GUN5 is to shift metabolism from protoporphyrin IX to chlorophyll biosynthesis [45]. Uroporphyrinogen III decarboxylase (UROD) is encoded by the HEME gene, and has catalytic activity involved in uroporphyrinogen III synthesized coproporphyrinogen III function; therefore, it plays an important role in chlorophyll synthesis and metabolism. It has been reported that transgenic tobacco lines with HEME-RNAi silenced resulted in a decrease in chlorophyll and heme content [46]. Previous studies have shown that, compared with normal green leaf tea trees, anthocyanin-rich and normal green leaf tea trees have different HEME expression levels, which may be the main reason for the variation of in chlorophyll content [47].

The synthesis and degradation of chlorophyll plays a key role in the formation of leaf color. For example, during the yellowing process of ginkgo leaves, a similar high expression level of chlorophyll degradation helps to accelerate the degradation of chlorophyll b to chlorophyll a, and finally leads to the production of ginkgo etiolated mutants without chlorophyll [48]. In the study of etiolated tea, it was found that relatively high expression levels of CLH were negatively correlated with chlorophyll content in etiolated leaves [12]. Under the different light qualities, the change of leaf color of ‘Huangjinya’ was closely related to CLH gene expression [49]. Some studies have also shown that the CAO gene can be induced by light in photosynthetic tissues, and the repression of the CAO gene will lead to the inhibition of the synthesis of chlorophyll b, the lowering of chlorophyll b content in the plant, and will result in the phenotype of pale green leaves [41].

**Figure 6:**
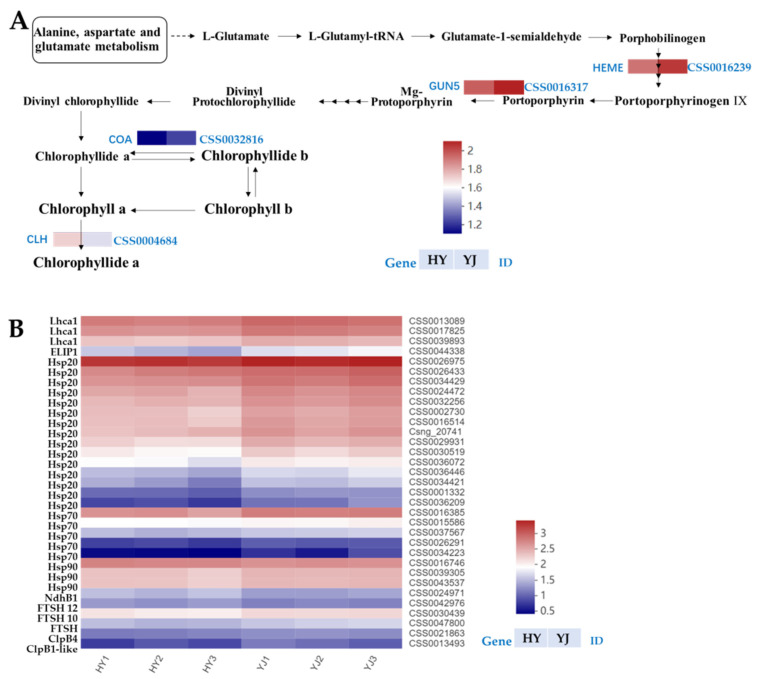
Expression profiles of DEGs involved in chlorophyll metabolism (**A**) and photosynthesis (**B**) photosynthesis-antenna proteins and photosynthetic proteins involved in endoplasmic reticulum pathway in ‘Yinghong 9’ and ‘Huangyu’. YJ is ‘Yinghong 9’, HY is ‘Huangyu’.

In this study, three DEGs involved in the chlorophyll synthesis pathway, namely HEME (CSS0016239), GUN5 gene (CSS0016317), and CAO gene (CSS0032816) were identified (Supplementary Table S9). Their expressions levels were significantly lower in ‘Huangyu’ than that of ‘Yinghong 9’, which indicated that the chlorophyll synthesis pathway was blocked in yellow leaves due to down-regulated expression of the above-mentioned three genes. In the chlorophyll degradation pathway, the expression level of chlorophyllase (CLH, CSS0004684) in ‘Yinghong 9’ was lower than that of ‘Huangyu’ leaves, indicating that in ‘Huangyu’ leaves, chlorophyll was degraded more than that of ‘Yinghong 9’, thereby lowering the chlorophyll levels (Figure 6A). Therefore, we speculated that the main reason for the lower chlorophyll content in ‘Huangyu’ leaves might be the result of the involvement of the above three genes. Similar results were found in some light-sensitive albino teas [12].

#### 3.2.2. The Down-Regulation of Light-Harvesting Protein and Chloroplast Proteases Decreases the Biosynthetic Capacity of Etiolated Tea

Pathways associated with photosynthetic proteins, including the photosynthesis-antenna protein pathway and the protein processing in the endoplasmic reticulum pathway. The plant photosynthetic system is closely related to the chloroplast. Previous studies have shown that the development of chloroplasts in etiolated mutants was abnormal, resulting in a significant down-regulation of different genes related to photosynthesis [36]. LHC is an important light-harvesting chlorophyll-a/b-binding protein in the plant photosynthetic system. Compared with normal green leaves, LHCA2, LHCA4, LHCB1, and LHCB3 were significantly down-regulated in yellow leaf tea, indicating that the photosynthetic activity decreased [36]. In this study, a total of three significantly differentially expressed LHCII genes, namely CSS0013089, CSS0017825, and CSS0039893, were screened in the photosynthesis-antenna protein pathway (Figure 6B). The expression levels of the three genes were higher in ‘Yinghong 9’ than that of ‘Huangyu’ (Figure 6B and Supplementary Table S9), which indicated that the photosynthetic capacity of ‘Yinghong 9’ was higher than that of ‘Huangyu’.

Protein processing in the endoplasmic reticulum is affected by external stress, which can interfere with the process of protein synthesis and denaturation. Heat shock proteins (HSPs) can also be used as protective proteins to maintain cellular homeostasis in plants [50]. HSPs have been observed to participate in leaf coloration by mediating chloroplast development in many plants. Inactivation of HSP100 family member clpC1 in *Arabidopsis* causes leaf yellowing and growth retardation by regulating chloroplast function. The transgenic *Arabidopsis thaliana* line that suppressed HSP70s appeared white, had a stunted leaf color phenotype, and had less or no chloroplast thylakoid membranes [51]. Multiple genes involved in protein processing pathways in the endoplasmic reticulum were significantly altered in etiolated cultivars, among HSPs (including Hsp20, Hsp40, and Hsp70), the indicator of stress levels, were significantly down-regulated in etiolated cultivars compared with normal green leaves. This suggests that the expression of HSPs and HSFs were repressed in etiolated cultivars, thereby affecting chlorophyll biosynthesis to regulate leaf coloration [36]. In this study, the CSS0026975 gene with the highest expression level was Hsp20, and the expression level of Yinghong 9 was higher than that of ‘Huangyu’. Four of the top twenty expressed genes were HSP genes, including HSP20, HSP70, and HSP90. Among the DEGs, except HSP90 (CSS0016746) whose expression level was up-regulated, the other HSPs were down-regulated, which indicated that most of the HSPs in ‘Huangyu’ were repressed, and thus decreased the biosynthetic capacity of ‘Huangyu’ (Figure 6B and Supplementary Table S9). The current research results was consistent with previous studies [36].

The ATP-dependent zinc metalloprotease FtsHs are a large family of chloroplast proteases involved in the degradation of unassembled proteins and the transport of photosynthetic protein complexes on the thylakoid membrane, and play important roles in chloroplast differentiation and photosynthetic protein complex formation [52,53]. Previous studies have found that FtsH2 or FtsH5 mutations in *Arabidopsis* can lead to leaf yellowing, while FtsH2 and FtsH8 double mutants have an albino phenotype, indicating that the FtsH subunit is an essential protein for chloroplast differentiation [53,54]. Wu et al. found that the content of FtsHs proteins (including FtsH2, FtsH8, and FtsH5) in the *Arabidopsis* variegated leaf mutant thf1 was significantly lower than that of the wild type, while the content of FtsHs protein in the pale green mutant clpR4-3 thf1 was significantly higher than that of the wild type [55]. In this study, three FtsHs DEGs were identified, namely CSS0022449, CSS0047800, and CSS0030439 (Supplementary Table S9), and the expression levels of these three genes were down-regulated in ‘Huangyu’.

In higher plants, the ATP-dependent Clp protease is mainly located in the chloroplast matrix, it can degrade a variety of matrix proteins, and is crucial to maintain the development and function of the chloroplast [56]. In this study, two Clp proteases were identified in the DEGs, namely chaperone protein ClpB4 (CSS0021863) and chaperone protein ClpB1-like (CSS0013493) (Figure 6B and Supplementary Table S9). Their expression levels were lower in ‘Huangyu’ than in ‘Yinghong 9’. This indirectly indicated that the chloroplast development of normal green leaf ‘Yinghong 9’ was better than that of the etiolated mutant ‘Huangyu’.

#### 3.2.3. Changes in Light-Induced Protein and Circadian Rhythm Promotes Etiolated Tea to Adapt Light Stress

Light and circadian rhythms can regulate the gene expression of magnesium chelatase subunits CHLI, CHLD, and CHLH. Circadian rhythms can not only regulate the expression of magnesium chelatase genes but also affect the activity of magnesium chelatase by regulating the relative concentrations of free ATP and magnesium ions in chloroplasts [57]. In addition, the circadian rhythm can regulate the expression of each subunit of plant magnesium chelatase with different regulatory modes. In this study, the rhythm regulation pathway was significantly enriched, including a total of 12 DEGs. In the etiolated mutant ‘Huangyu’, except for CSS0030421 and CSS0047294, the remaining 10 genes were down-regulated, which may lead to the difference in chlorophyll metabolism between ‘Yinghong 9’ and ‘Huangyu’ (Supplementary Table S8).

In the study of the light-sensitive etiolated tea mutant ‘Huangjinya’, it was found that the composition and activity of photosystem II (PSII) subunits were directly related to the yellowing phenotype. Studies have shown that the PSII subunit in ‘Huangjinya’ was more susceptible to high-light stress than in green leaves. A series of subunits in PSII and the light-harvesting chlorophyll protein complex are degraded when they are induced by high-light stress, resulting in the exposure of the D1 and D2 groups of the PSII reaction center to Deg protease and FtsH, and then they are degraded by Deg protease and FtsH. Eventually it leads to a decrease in chlorophyll concentration or the albino phenotype. Interestingly, these processes are reversible, and it leads to adaptations to high-light stress in the etiolated tea mutant [58].

Previous studies have shown that the function of ELIPs may be involved in the regulation of chlorophyll concentration in thylakoids, by inhibiting the entire chlorophyll biosynthetic pathway from the initial precursor tetrapyrrole 5-aminolevulinic acid. In this study, an early light-induced protein ELIP1 (CSS0044338) was significantly down-regulated in ‘Huangyu’ (Supplementary Table S9), and the result implied that chlorophyll synthesis and the accumulation of free chlorophyll was repressed in ‘Huangyu’.

In addition, among all DEGs, the expression level of chlorophyllase in etiolated leaves was significantly lower than that of normal green leaves; four DEGs involved in the carotenoid biosynthesis pathway were significantly enriched, and expression levels were down-regulated in etiolated mutant; they were beta-carotene hydroxylase 2 (BCH2, CSS0044082), 9-cis-epoxycarotenoid dioxygenase (NCED1, CSS0033791), 9-cis-epoxycarotenoid dioxygenase 1 (NCED1, CSS001521), and abscisic acid 8′-hydroxylase 4-like (CSS0004890) (Supplementary Table S7). This result was consistent with the lower carotenoid content in the ‘Huangyu’ mutant. Current results were also consistent with the expression profiles of carotenoid genes in several etiolated plants [59].

### 3.3. Transcription Factors May Determine the Differences in Chlorophyll Synthesis and Photosynthetic Capacity

The RADIALIS-like gene contains a typical SANT/MYB domain and belongs to the I-box-binding-MYB transcription factor family. This gene has a light response regulatory element I-box, so it may be involved in cell development and metabolite synthesis in photomorphogenesis. Previous studies confirmed that *Oryza sativa* RADIALIS-LIKE3 (*OsRL3*) promoted dark-induced leaf senescence and reduced the susceptibility to salt stress in rice. In the dark, *osrl3* null mutants exhibited a stay-green phenotype, with increased chlorophyll retention and photosynthetic capacity [60]. In this study, the RADIALIS-like (CSS0035656) transcription factor had the highest expression level among all transcription factors, and the expression level of RADIALIS-like in ‘Huangyu’ was higher than that of ‘Yinghong 9’. We speculated that it is related to the enhancement of the photosynthetic capacity and modifying to the lack of chlorophyll in the etiolated mutant ‘Huangyu’ (Supplementary Table S10).

Transcription factor HY5 is a leucine zipper (bZIP) family transcription factor, which can harvest light signals through different light-sensing signals, and transmit the light signals to downstream acting elements, thereby regulating plant growth and development [61]. HY5 is a critical factor involved in IAA and the chlorophyll synthesis pathway. Studies have found that HY5 is involved in the regulation of Chl biosynthesis downstream of GUN5 and HSP90 [62]. Studies have also revealed that both photomorphogenesis and nitrogen uptake, as well as assimilation in plants are regulated by the HY5 transcription factor, and these regulatory methods can be mediated by light [63]. The phenotypes of plant photomorphogenesis, such as elongation of seedling hypocotyl, and yellowing and de-etiolating of plants, are closely related to the expression of HY5. In addition, HY5 also initiates phytochromes, cryptochromes, and UV-B photoreceptors in photomorphogenesis downstream [64]. Additionally, HY5 is involved in plant chloroplast development and chlorophyll accumulation. HY5 acts as a central repressor in light signaling to enhance photomorphogenesis, of which POR and CAO are its potential regulatory target genes [65]. A recent study found that in ‘Xiangfeihuangye’ etiolated tea leaves, HY5, HemH, and CLH were up-regulated, while the HemA and POR genes were down-regulated, implying that chlorophyll synthesis was repressed due to increased expression of HY5, thus increasing the rate of catabolism [66]. While in this study, the changes of HY5 were not consistent with the above results, that was, the expression level of HY5 (CSS0048476) in the etiolated mutant ‘Huangyu’ was lower than that of the normal green leaf ‘Yinghong 9’, while the expression level of CLH was similar to ‘Yinghong 9’ (Supplementary Table S10). Considering that CLH can degrade chlorophyll, it has negative impacts on the accumulation of chlorophyll. Therefore, in etiolated ‘Huangyu’ leaves, the low expression levels of HY5 are not advantageous for chlorophyll synthesis, and due to the enhanced catabolism by CLH, the content of chlorophyll is considerably lower than that of ‘Yinghong 9’.

In addition, among the highly expressed transcription factors, ethylene-responsive transcription factor RAP2-12-like, Auxin-responsive protein IAA9, ETHYLENE INSENSITIVE 3-like 1 protein, and so on, were up-regulated in ‘Huangyu’, while the expression levels were correspondingly lower in ‘Yinghong 9’. The reason may be that when induced by IAA and other biological signals, photosynthetic capacity was enhanced in etiolated tea to modify to the environment stress. The light-responsive PAP1-like MYB transcription factor (MYB75) plays an important role in anthocyanin biosynthesis in tea [67,68]. R2R3-MYB TFs can directly bind to the promoter regions of structural genes to regulate anthocyanin biosynthesis [69]. In addition, the study has also found that MYB14 was a bifunctional transcription factor involved in regulation of proanthocyanidin accumulation, and could convert procyanidins from insoluble to soluble forms. In this study, several differential expression R2R3-Myb transcription factors were all down-regulated in ‘Huangyu’ leaves, including Myb75 (PAP1) (CSS0046378), MYB14 (CSS0014476), MYB123 (CSS0036919), and so on (Supplementary Table S10). Since the light-harvesting complex (LHC-II) and light-harvesting antenna protein were severely damaged in ‘Huangyu’, these down-regulated light-responsive transcription factors may contribute to the less accumulation of polyphenols and anthocyanins in etiolated leaves.

### 3.4. Difference of Leaf Colors Lead to Differences in Metabolic Direction and Accumulation of Flavor Components

The flavanols and flavonols not only form the flavor and quality of tea but also have contributions to the appearance of the finished tea. Chalcone, aurone, and luteolin are yellow pigments that contribute to the coloration of yellow flowers [70,71]. The mutations of chalcone isomerase (CHI) and dihydroflavonol 4-reductase (DFR) lead to the accumulation of chalcone-glucoside, and produce yellow flowers in carnations [72]. In addition, the knockdown of DFR and F3H also repressed anthocyanin biosynthesis and produced yellow flowers in snapdragon flower. Similarly, the total catechins in the etiolated mutant ‘Huangjinya’ were significantly lower than in normal green leaf tea [24], which indicated that catechin biosynthesis was repressed in etiolated tea. Since the expression levels of genes involved in polyphenols and flavonoid biosynthesis in the ‘Huangjinya’ mutant were negatively correlated, the light-induced expression genes involved in flavonoid biosynthesis would repress the accumulation of polyphenols, which can partly explain the formation mechanism of the ‘Huangjinya’ leaf color [20].

Most studies found that the concentration of flavonoids was significantly lower in the albino tea due to impaired photosynthesis and chlorophyll metabolism [35,73]. However, not all gene expression tendencies involved in flavonoid biosynthesis in etiolated tea were consistent. For example, the transcript levels of most flavonoid genes were higher in the etiolated leaves of ‘Huangjinya’ than in normal green leaves [20]. The expression level of FLS in the etiolated ‘Rougui’ mutant was significantly higher than that of normal green leaves [74]. It was found that most flavonoid genes were down-regulated in the etiolated ‘Xiangfeihuangye’ mutant, while flavonoid glycosides were accumulating continuously [66]. It may be an alternative strategy for plants to alleviate oxidative stress [75]. In addition, studies have also found that the metabolic flux shifts from catechin to quercetin in etiolated tea mutants, which results in higher resistance to light damage. Correspondingly, CHS, CHI, F3H, F3′5′H, DFR, and FLS may be ‘turning points’ for the metabolic flux redirection in etiolated tea [32]. The expression levels of the related genes involved in flavonoid biosynthesis were down-regulated and the target protein content of ANR and CHI was also reduced. These results can provide insights into the catechin biosynthesis mechanism in variegated tea phenotypes [12].

In this study, in addition to the obvious differences in phenolic acids between ‘Yinghong 9’ and ‘Huangyu’, the contents of flavonoids and tannins were also higher in ‘Huangyu’ than in ‘Yinghong 9’. Interestingly, although photosynthesis was partly suppressed in etiolated tea, the changes had less effects on the flavonoid metabolism in tea, and even the anabolism of flavonoids has been strengthened. Previous studies have confirmed that the metabolic flux shifted to flavonoid and carotenoid pathways in the ‘Yujinxiang’ yellow leaf mutant to varying extents compared to the normal green cultivar. Changes in metabolic flux may enhance the production of the antioxidant quercetin or quercetin glycosides rather than catechin biosynthesis [32].

**Figure 7:**
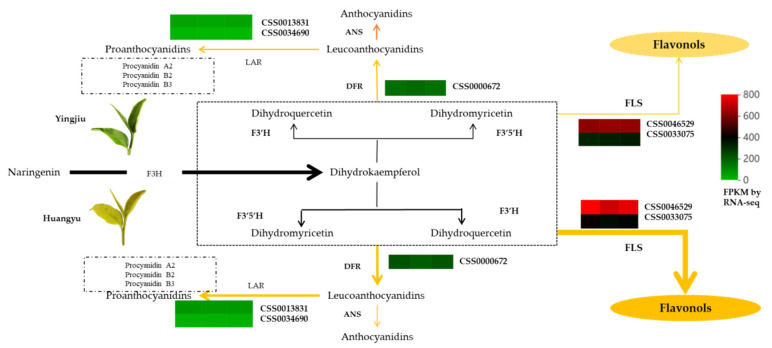
DEGs involved in flavonoid pathway and expression levels in ‘Yinghong 9’ and ‘Huangyu’. The heatmap is created according to the average expression levels of related biosynthetic genes based on the FPKM value by RNA-seq. Green indicates low expression levels, and red indicates high expression levels. ANS, anthocyanidin synthase; DFR, dihydroflavonol reductase; F3′5′H, flavonoid 3′,5′-hydroxylase; F3H, flavanone-3-hydroxylase; F3′H, flavonoid 3′-hydroxylase; FLS, flavonol synthase; LAR, leucoanthocyanidin reductase. Heavy lines indicate the enhancement of metabolic flux in ‘Huangyu’, and thin lines indicate the attenuation of metabolic flux in ‘Yinghong 9’.

According to the KEGG enrichment analysis results, phenylpropanoid biosynthesis and flavonoids pathways were significantly enriched, including thirty and six DEGs, respectively. Among DEGs, except for six lower expression level genes (FPKM = 1–35) involved in phenylpropanoid biosynthesis pathway, the remaining thirty DEGs were up-regulated, of which two CsFLS (CSS0046529, CSS0033075) had the highest expression levels in ‘Huangyu’, followed by CsDFR (CSS0000672). Two LAR genes (CSS0013831 and CSS0034690) were also up-regulated in ‘Huangyu’ (Figure 7 and Supplementary Table S6B). FLS and DFR are critical genes in flavonol metabolism; the disequilibrium of FLS and DFR expression levels will lead to different levels of anthocyanin and flavonols accumulation in tea [76]. In this study, although the content of chlorophyll was lower in ‘Huangyu’, it had less effects on flavonoid metabolism. The reason might be that in order to avoid ultraviolet damage in light stress, the metabolic flux shifted to more synthesis in the etiolated mutant. Therefore, flavonoid content was less variable in etiolated tea. The results of this study were consistent with previous finding [20,32,66,74]. Although some studies have found that the expression levels of genes involved in flavonoid synthesis were decreased in etiolated rice leaves [77], it was inconsistent with the results of this study. The reason might be that the metabolic mechanism of rice was very different from that of *theaceae* species.

As previously mentioned, among the important differential metabolites in ‘Huangyu’, quercetin-7-*O*-rutinoside-4′-*O*-glucoside and kaempferol-3-*O*-(6′-rhamnosyl-2′-glucosyl-glucoside) were significantly down-regulated, and the remaining flavonoid metabolites were up-regulated. The content of some flavonols in ‘Huangyu’ was higher than that of ‘Yinghong 9’. Whereas flavonol glycosides play important roles in the formation of tea flavor [78,79], the lower ratio of kaempferol-3-glucoside to kaempferol-3-galactoside is suitable for the manufacturing of black tea [80]. In this study, the content of kaempferol-3-glucoside in ‘Yinghong 9’ was 1.5-fold that of ‘Huangyu’, and the ratio of kaempferol-3-glucoside to kaempferol-3-galactoside was lower (Supplementary Table S3A). The results indicated ‘Yinghong 9’ and ‘Huangyu’ have differences in manufacturing suitability.

## 4. Materials and Methods

### 4.1. Plant Materials

Fresh tea leaf material, consistent with a plucking standard of one bud and two leaves was collected in August 2020 at the same experimental tea garden (22°56′ N, 112°29′ E) of Guangdong Xiangshun Xiangwo Chancha Ecological Agriculture Co., Ltd. (Xinxing County, Yunfu, China). Fresh leaves of the etiolated mutant (‘Huangyu’) were collected from the mutant plants, which were grafted on the parent plants (’Yinghong 9′). The collected fresh tea leaves were immediately fixed in liquid nitrogen. The samples were ground into a powder and stored at −80 °C for pigment determination, metabolome analysis, and transcriptome sequencing.

### 4.2. Determination of Tea Polyphenol, Chlorophyll, and Carotenoids

#### 4.2.1. Determination of Polyphenols Content in Tea

The polyphenols content of tea was determined by the spectrophotometric method. The frozen ground samples (1.5 g) were extracted by 125 mL boiling water in a 37 °C water bath for 45 min. The absorbance of the tea extract with phosphate buffer and dyeing solution (containing 3.6 × 10^−3^ M FeSO_4_ and 3.5 × 10^−3^ M potassium sodium tartrate, KNaC_4_H_4_O_6_) was measured at 540 nm by using a spectrophotometer (Shanghai UNICO Instrument Co., Ltd., Shanghai, China). Distilled water containing phosphate buffer and FeSO4 dyeing solution was set as a control. Total content of tea polyphenols was calculated according to a previous report [81]. The content of total catechins was determined based on the reaction of vanillin–HCl. Briefly, 2.5 mL of tea extract was mixed with 6.5 mL of 1% (*w*/*w*) vanillin solution and 15 mL of 4% (*v*/*v*) HCl (both dissolved in ethanol). Reaction was left for 30 min at 30 °C, and finally, the absorbance of the sample was measured at 505 nm. Catechin was used as the standard (0 to 200 mg·L^−1^), and the results were expressed as milligrams of catechin equivalents per liter of sample (mg·L^−1^).

#### 4.2.2. Measurement of Chlorophyll and Carotenoids

An aliquot of freeze-dried and powdered samples (0.25 g) from three independent biological replicates was mixed with 12.5 mL of 95% ethanol (*v*/*v*) and incubated in darkness for 24 h. The extracts were analyzed using micro-plate absorbance reader (Sunrise, TECAN, Männedorf, Switzerland) at 665 nm for chlorophyll a (Chl a), 649 nm for chlorophyll b (Chl b), and 470 nm for total carotenoids. Total chlorophyll (Chl mg/g) content, Chl a/b ratio, and carotenoid content were calculated according to the method and formula of Song and Tian [20,82]. The contents of chlorophyll and carotenoid were estimated against mg/g fresh weight (FW).

### 4.3. Analysis of Metabolite Compounds in ‘Yinghong 9’ and ‘Huangyu’

The lyophilized samples were ground with a laboratory mill (Retsch, Verder group, Haan, Germany). We used 1.2 mL of 70% (*V*/*V*) methanol solution to extract the metabolites from 100 mg of ground samples. The extracted metabolic compounds were analyzed using a UPLC-MS/MS system (SHIMADZU, Kyoto, Japan). The chromatographic solvent system and chromatographic conditions were performed according to the protocol described by Zhou [76]. The chromatographic separation was conducted on an Agilent UPLC column SB-C18 (1.8 µm, 2.1 mm × 100 mm) at 40 °C. The mobile phase consisted of solvent A (pure water with 0.1% formic acid) and solvent B (acetonitrile with 0.1% formic acid). Sample measurements were performed with a gradient program that employed the starting conditions of 95% A, 5% B. Within 9 min, a linear gradient to 5% A, 95% B was programmed, and a composition of 5% A, 95% B was kept for 1 min. Subsequently, a composition of 95% A, 5.0% B was adjusted within 1.10 min and kept for 2.9 min. The flow rate of the mobile phase was 0.35 mL/min; the injection volume was 4 μL. Metabolic compounds identification and quantification were based on the primary and secondary spectral data annotated on MVDB database V2.0 (Metware Biotechnology Co., Ltd., Wuhan, China) and public databases such as MassBank, HMDB, KNAPSAcK, and METLIN [15]. The orthogonal partial least squares discriminant analysis (OPLS-DA) was carried out using the R package with the identified metabolites [83]. Significantly differential metabolites between ‘Yinghong 9’ and ‘Hongyun’ were determined when its variable importance in the project (VIP) ≥ 1.0 and fold change ≥ 1.0 or *p*-value < 0.05.

### 4.4. Isolation and Analysis of Carotenoid Compounds

An aliquot of freeze-dried, ground samples (100 mg) was mixed with 1 mL of extraction solution (acetone:n-hexane:ethanol, 2:1:1, *v*/*v*/*v*) with 0.01% BHT (butylated hydroxytoluene, g/mL). The sample was extracted by vortexing at 25 °C for 20 min. The supernatant was collected by centrifugation (4 °C, 12,000 rpm, 5 min) and then was dried by blowing nitrogen. The qualitative and quantitative analyses of carotenoids in ‘Yinghong 9’ and ‘Huangyu’ were conducted by using UHPLC-APCI-MS/MS. Identification and quantification of carotenoid compounds were performed at Metware Biotechnology Co., Ltd. (Wuhan, China), and chromatographic conditions were in accordance with the previous report [84]. The content of each carotenoid compound detected in the samples was calculated by using the equations of standard curves (see the attached Supplementary Table S1 for the standard curves).

### 4.5. RNA-seq Analysis

Total RNA of tea leaf samples was extracted, sequenced, and analyzed as described previously [76]. In brief, 1.0 g of frozen sample was extracted by using TIANGEN RNAprep Pure Plant Kit (DP441, TIANGEN Biotech Co., Ltd., Beijing, China). The cDNA libraries were prepared and sequenced on the Illumina Novaseq6000 platform at Biomarker Technologies Corporation (Beijing, China). The raw data have been deposited in National Genomics Data Center, China, National Center for Bioinformation with an accession number CRA008238. The raw reads were trimmed, and quality controlled using SeqPrep software to get clean data. The remaining clean reads were then mapped onto the *Camellia sinensis* var. *sinensis* (CSS) genome (http://tpdb.shengxin.ren, accessed on 1 March 2022) to obtain mapped reads and quality assessment using HISAT2. Based on the selected reference genome sequence, the mapped reads were assembled using StringTie software and then compared with the original genome annotation information to discover new transcripts and new genes. All obtained unigenes were aligned with the non-redundant (NR) database, with E < 1 × 10^−5^ as the threshold, and functional annotation was performed by the BLAST algorithm. At the same time, the functions of unigenes in the public databases of Swiss-Prot, Pfam, COG, GO, and KEGG were annotated, and their functional classification and biological pathways were analyzed and predicted. Gene expression levels were assessed using the fragments per kilobase of transcript per million mapped reads (FPKM) method. Differential expression analysis was performed using DESeq2 (v 1.6.3, Bioconductor, Boston, MA, USA) software, and DEGs were screened by false discovery rate (FDR) and fold change (FC) method (FC ≥ 1.5, FDR < 0.01). All DEGs were subjected to GO functional annotation and KEGG enrichment analysis using GOseq (v 3.16, Bioconductor, Boston, MA, USA) and KOBAS (v 3.0, Peking University, Beijing, China) software, respectively. Transcription factors were identified from screening DEGs.

### 4.6. Quantitative Real-Time PCR Validation

To verify the results of RNA-seq, nine highly expressed differential genes were selected for expression level verification. Fluorescent quantitative primers were designed by Premier 5.0 software (Supplementary Table S2). β-actin was used as the reference gene, and the primers were F: GCCATCTTTGATTGGAATGG and R: GGTGCCACAACCTTGATCTT. A total of 1 µg of extracted total RNA from tea samples was used to synthesize first-strand cDNA with the FastKing One Step (SYBR Green) cDNA Synthesis Kit (FP313, TIANGEN Biotech Co., Ltd., Beijing, China) as described in the protocol. The PCR amplification program was as follows: 95 °C (30 s) followed by 39 cycles at 95 °C (10 s), 50 °C (15 s), and 72 °C (30 s). All sample analyses were conducted with three independent biological replicates. The 2^−∆Ct^ method was used to calculate the relative genes expression levels of qRT-PCR results [85].

### 4.7. Data Analysis and Figure Presentation

The experimental data were analyzed by Excel 2010 (Microsoft, Redmond, WA, USA); the Pearson correlation coefficients between samples were calculated by SPSS software v24.0 (SPSS Inc., Chicago, IL, USA). The significant analysis was performed by the Duncan test. Some figures and tables related to metabolites and transcriptomes were prepared on the BMKcloud platform (https://international.biocloud.net/, accessed on 29 March 2022). The heatmap of carotenoids was presented by using TBtools v1.098765 (for Windows 64-bits, South China Agricultural University, Guangzhou, China) [86].

## 5. Conclusions

In this study, we investigated biochemical differences between ‘Yinghong 9’ and its mutant ‘Huangyu’. The results showed that the lower chlorophyll content as well as the lower ratio of chlorophyll to carotenoids contributes to the yellowing leaf color of ‘Huangyu’. A total of 46 differential metabolites were identified by metabolomic analysis, of which 26 were up-regulated and 20 were down-regulated in ‘Huangyu’. Furthermore, compared with the ‘Yinghong 9’ normal green leaves, the content of zeaxanthin was significantly higher in etiolated ‘Huangyu’. A total of 1009 DEGs were screened using RNA-seq analysis, of which four DEGs were involved in chlorophyll synthesis and degradation, except the CLH gene, and 34 DEGs were involved in photosynthesis-related proteins (including 23 HSPs genes) which were down-regulated in ‘Huangyu’. The lower chlorophyll content in ‘Huangyu’ leaves might be attributed to the lower expression levels of HEME, GUN5, and CAO involved in chlorophyll synthesis and higher expression levels of CLH in chlorophyll degradation. It implies that changes in the expression of genes and transcription factors involved in chlorophyll synthesis and chloroplast biogenesis led to the etiolated leaf color. Not only FLSs, DFR and LARs were all up-regulated in ‘Huangyu’, but also the total expression level of the FLS gene was 3.4-fold higher than that of DFR and LARs. We deduced that the disequilibrium of expression levels of the FLS, DFR, and LAR in etiolated tea leaves resulted in shifting metabolic flux to the accumulation of flavonols in rather than catechins. Our results provide novel insights into the metabolic and transcriptional mechanisms in the etiolated tea mutant.

## Data Availability

The raw sequence data reported in this paper have been deposited in the Genome Sequence Archive in National Genomics Data Center, China National Center for Bioinformation/Beijing Institute of Genomics, Chinese Academy of Sciences (GSA: CRA008238) that are publicly accessible at https://ngdc.cncb.ac.cn/gsa, accessed on 21 September 2022.

## Supplementary Materials

The supporting information can be downloaded at: https://www.mdpi.com/article/10.3390/ijms232315044/s1.
